# Chronic Social Stress Time-Dependently Affects Neuropathic Pain-Related Cold Allodynia and Leads to Altered Expression of Spinal Biochemical Mediators

**DOI:** 10.3389/fnbeh.2017.00070

**Published:** 2017-05-09

**Authors:** Glenn-Marie Le Coz, Julien Genty, Fernand Anton, Ulrike Hanesch

**Affiliations:** Laboratory of Neurophysiology and Psychobiology, Institute for Health and Behavior, University of LuxembourgLuxembourg, Luxembourg

**Keywords:** neuropathy, social stress, cold sensitivity, mechanical sensitivity, biochemical pathways

## Abstract

Clinical data have shown that chronic exposure to stress may be accompanied by an enhancement of inflammation-related pain sensitivity. In this context, little is however known on the impact of stress on neuropathic pain. In the present study we addressed this issue by combining the chronic constriction injury (CCI) model with an ongoing social stress (OSS) paradigm. Cold plate and von Frey tests were performed in 48 rats divided into four groups: OSS exposed to OSS, CCI subjected to chronic nerve constriction, OSS+CCI with a combination of neuropathy and stress and CON, a control group lacking any manipulation. While we did not observe any stress-related differences in mechanical sensitivity throughout the observation period, CCI rats were more sensitive to cold stimulation than OSS+CCI in the initial phase of neuropathy. A switch was observed at a later stage, leading to a hypersensitivity of the OSS+CCI compared to the CCI rats. At this time point we investigated the spinal mRNA expression of neuron and glia related molecules potentially involved in neuropathic pain and stress. The combination of psychosocial stress and neuropathic pain seemed to enhance glial cell activation, pro-inflammatory cytokine and neurotrophic factor mRNA levels, rather than glutamatergic transmission. Our data show that long lasting social stress may lead to time-dependent alteration of neuropathy-related cold pain sensitivity while mechanically-induced pain remains unchanged.

## Introduction

While exposure to stress may lead to the classically described phenomenon of stress-induced analgesia (SIA, for review see Butler and Finn, 2009), increasing amounts of data suggest that it may under certain conditions also lead to an enhancement of pain, denominated as stress-induced hyperalgesia (SIH, see Jennings et al., 2014). In this context, research has laid a more pronounced emphasis on inflammatory as compared to neuropathic pain, a clinical entity that remains difficult to treat (Finnerup et al., 2010). In this context comorbidities between chronic stress and pain may also have to be considered (Sharp and Harvey, 2001; Shipherd et al., 2007). Recent preclinical studies have also shown that exposure to chronic stress may lead to an exacerbation of pain sensitivity (Shi et al., 2010; Bravo et al., 2013; Burke et al., 2013).

Several biochemical pathways and mediators known to be involved in the processing of stress may also play a major role in regulating neuropathic pain. Stressors such as early life stress (Burke et al., 2013) and stress-related catecholamine release (O’Connor et al., 2003; Johnson et al., 2005) have e.g., been described to lead to neuroinflammatory reactions encompassing the peripheral and central release of pro-inflammatory cytokines like IL-1β and IL-6. These substances have been shown to play a crucial role in the enhancement of nociceptive processing (for review, see Watkins et al., 2001). Another major player in the context of the present article is glutamate, the principal excitatory neurotransmitter in the central nervous system (CNS). Glutamatergic transmission is significantly modulated by stress-related release of corticosteroids. On one hand stress may exacerbate neuropathic pain via glucocorticoid-dependent enhancement of NMDA receptor activation and glutamate release (Imbe et al., 2006; Alexander et al., 2009; Popoli et al., 2012). On the other hand it has recently been shown that corticosterone may also mediate analgesia via the spinal production of neuroactive metabolites that enhance GABAergic inhibitory transmission (Zell et al., 2015). With regard to neurotrophic factors, stress-induced release of nerve growth factor (NGF) and brain-derived neurotrophic factor (BDNF) is involved in the neurobiological alterations underlying increased vulnerability to disease in humans (Cirulli and Alleva, 2009); but NGF is also a critical mediator for the enhancement and maintenance of inflammatory (Woolf et al., 1994; Watson et al., 2008) and neuropathic pain (Santos et al., 2012). Recently, dynamic epigenetic regulations of the glial cell line-derived neurotrophic factor (GDNF) promoter in the nucleus accumbens (NAc) also have been shown to play important roles in determining both the susceptibility and the adaptation responses to chronic stressful events (Uchida et al., 2011). In the context of the present article, GDNF has been demonstrated to have a beneficial effect in neuropathic pain states (Boucher et al., 2000).

Stress-related activation of the HPA axis leads to the release of glucocorticoids (GC). Alterations of adrenocortical reactivity have been shown to impinge on the further coping with stress (Heim et al., 2000) as well as on nociceptive processing (Straub et al., 2002; Geiss et al., 2005; Le Coz et al., 2014a,b). In a model of persistent stress in rodents, Schmidt et al. (2007) highlighted lasting adaptations of the HPA axis that could modify the biochemical cascades described above (i.e., glial cell activation, pro-inflammatory cytokines release, neurotrophic factors and glutamatergic transmission).

The effects of stress on the processing of nociception and pain may depend on a host of factors including the kind of stress (physical or psychological), the intensity as well as the temporal characteristics (Jennings et al., 2014). In order to take into account that humans are mainly exposed to persistent or intermittent psychosocial stress in modern societies we used a psychosocial stress paradigm initially introduced in mice by Schmidt et al. (2007) but inspired by previous models in rats (Mormède et al., 1990).

Taking into account the findings and considerations described above, we hypothesized that long lasting social stress may enhance pain sensitivity related to chronic constriction injury (CCI), a well-established model of neuropathic pain (Bennett and Xie, 1988). Matching alterations of biochemical pathways concomitantly involved in the processing of stress and pain should provide clues to potentially involved mechanisms. For the biochemical analyzes we focused on the spinal cord, which constitutes the first relay station and site of complex processing and gating of nociceptive information and contains all the mentioned stress- and pain-related mediators (Golovatscka et al., 2012).

## Materials and Methods

### Animals

Experiments were performed in 48 male Sprague Dawley rats being 3 weeks old and weighing 48–86 g at arrival. We only used male rats to avoid estrogen-related effects on glucocorticoid release. The animals (Harlan Laboratories, Netherlands) were housed three per cage in a temperature-controlled room (20–22°C) under a 12 h day-night cycle. Food and water were provided *ad libitum*. Starting 1 week before initiation of the behavioral experiments, the animals were handled daily and habituated to the testing room and devices.

All animal experiments were carried out in accordance with the European Communities Council Directive of September 22, 2010 on the protection of animals used for scientific purposes (2010/63/EU). The animal procedures were approved by the Animal Care and Use Committee of the University of Luxembourg.

### CCI Surgery

For CCI surgery at an age of 8 weeks, the animals were anesthetized with isoflurane (4.3% for induction and 2.5% for maintenance) using an anesthesia unit (Univentor 400, Zejtun, Malta). The right sciatic nerve was exposed at the level of the thigh, as described in the classical model by Bennett and Xie (1988) and three loose ligatures using natural chromic gut 4–0 (Stoelting Europe, Dublin, Ireland) were placed around the nerve with an interspace of 1 mm. The muscle layers were stitched together with 4–0 silk sutures and the skin layer was closed with surgical skin staples.

### Experimental Protocol

**Figure 1:**
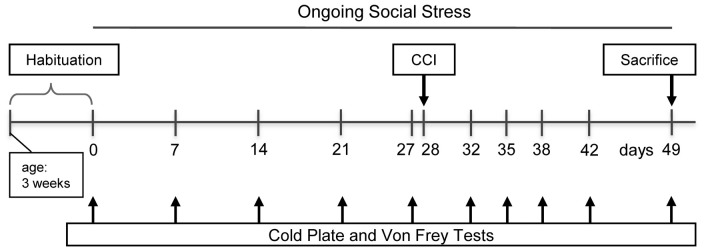
**Timeline of the experimental protocol**. Testing paradigms and stress induction were initiated at the age of 4 weeks.

The rats were divided into four groups: the control group CON undergoing no manipulation (*n* = 12), the ongoing social stress group (OSS) being exposed to the stress procedure (*n* = 12), CCI subjected to chronic constriction injury (*n* = 12), and OSS+CCI combining the CCI surgery with chronic social stress (*n* = 12). The course of the experiment was as follows (see Figure 1): 4 weeks before the CCI surgery in the respective groups (day 0), the chronic stress protocol was started in the OSS and OSS+CCI rats and continued until the end of the experiment at day 49. The social stress therefore was performed during adolescence and early adulthood in the age ranging from 4 to 11 weeks and had a total duration of 7 weeks. The cold plate and Von Frey tests were performed once a week (days 0, 7, 14, 21 and 27) in all groups in the morning hours. At day 28, the CCI and OSS+CCI rats underwent the CCI surgery. Testing for cold and mechanical sensitivity went on at days 32, 35, 38, 42 and 49 in all groups. Animals were sacrificed after the experiments at day 49 in the early afternoon and the spinal cord was removed and processed for qPCR.

### Chronic Social Stress Protocol

Rats were placed three per cage at their arrival and were enabled to habituate to this situation for 1 week before starting the stress protocol. The chronic social stress paradigm consisted in changing the composition of the adolescent cage-mates twice a week starting 4 weeks before the eventual CCI surgery until 21 days post CCI. A group size of 12 rats was chosen to avoid a recurrent composition of the cage-mates (Schmidt et al., 2007).

### Cold Plate Test

The cold plate (Hot/cold plate, Ugo Basile, Varese, Italia) was used to assess the sensitivity to thermal stimuli. A temperature of 5°C has been shown by Jasmin et al. (1998) to be optimal for testing cold hyperalgesia. Rats were placed three times on the cold plate for 3 min. The three repetitions were separated by a time out of 10 min in their respective home cages. The paw lifts were counted during the 3 min and the mean of the three sessions was calculated for each rat. Finally the mean for each group and day was calculated.

### Von Frey Monofilament Test

We used the Von Frey test to measure mechanical allodynia/hyperalgesia. Rats acclimated 10 min in Plexiglas® cages with wire mesh bottoms before the tests. Each monofilament (OptiHair, MarstockNervTest, Germany) was placed perpendicularly onto the midplantar region of the hind paw and pressure was increased until the point of deflection of the filament was reached. The ascending and descending method of limits was applied with forces ranging from 8 mN to 256 mN in 11 logarithmic steps to determine pain thresholds (Chaplan et al., 1994). Ascending and descending series were repeated three times and the thresholds obtained in the respective series were averaged for each paw. We then referred the ipsilateral paw values to the results of the contralateral side (control) set at 100%.

### Tissue Sample and RNA Collection

At day 49 (= 21 days post surgery), rats were deeply anesthetized with isoflurane and decapitated. Levels L4/L5 of the spinal cord were removed, the right (ipsilateral) side separated from the left (contralateral) side and the total RNA was extracted with the Invitrap Spin Tissue RNA Microkit (Invitek, Germany). The RNA concentration was determined by measuring the absorbance at 260 nm, using a Nanodrop ND-2000 spectrophotometer (Thermo Fisher Scientific, Wilmington, DE, USA).

### Reverse Transcription and Real-Time qPCR

Total RNA (500 ng) was reverse transcribed into cDNA using the ImProm-II Reverse Transcription System (Promega Corporation, Madison, WI, USA). Real-time PCR reactions were performed from 10 ng of cDNA with a CFX-96 thermocycler (Bio-Rad Laboratories, Nazareth, Belgium) using SYBR green Supermix PerfeCTa (95053-02K, Quanta Biosciences, Gaithersburg, MD, USA). The primers used for the reference gene, β-actin, and the genes of interest are presented in Table 1.

**Table 1:** **Sequences of primers used in this study**.

Name	Accession	Sequence
b-actin	NM_031144	5′ GCT GAG AGG GAA ATC GTG CGT GAC 3′
		5′ GGA GGA AGA GGA TGC GGC AGT GG 3′
Iba1	NM_017196	5′ TCC CAT CCA ACC TCT CTT CC 3′
		5′ GCA GCC TCA TCG TCA TCT C 3′
GFAP	NM_017009	5′ TGA GTC GCT GGA GGA GGA G 3′
		5′ GCT GTG AGG TCT GGC TTG G 3′
IL-1β	NM_031512	5′ GTT GAA TCT ATA CCT GTC CTG TG 3′
		5′ TGG TCT TGA CTT CTA TCT TGT TG 3′
IL-6	NM_012589.1	5′ CCA GAG TCA TTC AGA GCA ATA C 3′
		5′ CTT CTC CAT TAG GAG AGC AT 3′
GDNF	NM_019139.1	5′ GTG TTG CTC CAC ACC GCG TCT 3′
		5′ GGT CTT CGG CGG GCG CTT C 3′
NGF	NM_001277055.1	5′ CAC GGA CAT CAA GGG CAA GGA 3′
		5′ GCT CGG CAC TTG GTC TCA AA 3′
EAAT3	NM_013032.3	5′ TCA TAG TCG GGA AGA AC 3′
		5′ AGC GGA ATG TAA CTG GAA GG 3′
NR1	NM_001270607	5′ GGT TGC GTG GGC AAC ACC AA 3′
		5′ CCG TCC GCA TAC TTA GAA GA 3′
NR2a	NM_012573.3	5′ CAG ATA ACA ATA AGA ACC ACA AG 3′
		5′ AAC ATC GCT ACA GTC CTT 3′

The steps consisted of one cycle of 3 min at 95°C and 40 cycles of amplification (10 s at 95°C, 30 s at 61°C). All samples were run in triplicate. Relative expression was estimated using the ∆∆Ct-method with β-actin as the reference. Threshold cycle values (Ct) were used to compute the amount of target gene mRNA in relation to the reference gene mRNA (β-actin). ∆Ct represents the difference between the number of cycles that were necessary to detect the PCR products of the target and the reference genes. The ∆∆Ct indicates the difference between the ∆Ct of the individual groups (CCI, OSS, OSS+CCI) and the ∆Ct of the control (CON) animals. The data were then expressed as 2^−∆∆^^CT^. The presented data are the mean values of the ipsilateral side for each group.

### Data Analysis

Statistical analyzes for the behavioral tests (cold plate and Von Frey) were carried out using a two-way (time × condition) analysis of variance (ANOVA) followed by a Tukey’s multiple comparison *post hoc* test to check for differences between groups. Data are presented as mean ± SEM. The qPCR data were analyzed by using one-way ANOVA followed by Scheffé’s multiple comparison *post hoc* test. They are presented as mean ± SD. A summary of the statistical analysis is given in Table 2.

**Table 2:** **Analysis of variance (ANOVA)—summary of *F*-values of the behavioral (two-way) and biochemical (one-way) studies**.

Two-way ANOVA
	**Interaction**	**Time**	**Condition**
Cold plate test	*F*_(27,425)_ = 38.79	*F*_(9,425)_ = 113.80	*F*_(3,425)_ = 278.20
Von Frey test	*F*_(27,437)_ = 40.63	*F*_(9,437)_ = 118.06	*F*_(3,437)_ = 360.46
	*p* < 0.0001 for all values			
**One-way ANOVA**
GFAP	*F*_(3,20)_ = 6.00	*p* = 0.004	
Iba1	*F*_(3,20)_ = 19.65	*p* < 0.0001	
IL-1β	*F*_(3,20)_ = 32.27	*p* < 0.0001	
IL-6	*F*_(3,19)_ = 16.38	*p* < 0.0001	
GDNF	*F*_(3,18)_ = 35.11	*p* < 0.0001	
NGF	*F*_(3,18)_ = 11.39	*p* < 0.0001	
NR1	*F*_(3,18)_ = 9.27	*p* = 0.001	
NR2a	*F*_(3,18)_ = 3.25	*p* = 0.046	
EAAT3	*F*_(3,18)_ = 6.36	*p* = 0.004	

The level of significance was defined as *p* < 0.05. Statistical tests were performed with IBM SPSS Statistics version 21 (IBM corporation, Somers, NY, USA).

## Results

### Development of Cold Hypersensitivity Following CCI Surgery and Chronic Social Stress

**Figure 2:**
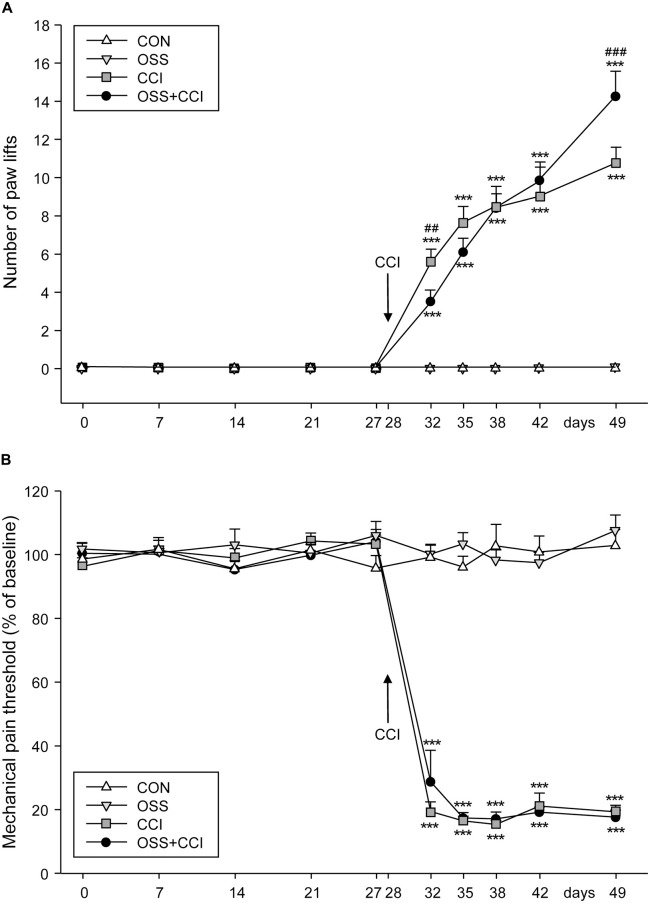
**Effect of ongoing social stress (OSS) and chronic constriction injury (CCI) on thermal pain sensitivity measured with the cold plate test and on mechanical allodynia/hyperalgesia. (A)** The number of right paw lifts (ipsilateral to constriction injury) per 3 min is expressed as mean ± SEM per group per day. In the 28 days preceding the CCI surgery, no paw lifts were observed in the four groups (*n* = 12 per group): CON (no manipulation, white triangles), OSS (exposed to ongoing social stress, light gray triangles), CCI (subjected to constriction injury, dark gray squares) and OSS+CCI (constriction injury under social stress conditions, black circles). After CCI surgery at day 28, cold hypersensitivity developed in the CCI and OSS+CCI groups with CCI being more sensitive in the first days and less sensitive after 49 days post-surgery than OSS+CCI. **(B)** Paw withdrawal response was measured by the Von Frey test. The CON and OSS groups did not display any differences in pain sensitivity compared to baseline during the entire experiment. The CCI and OSS+CCI rats demonstrated an important increase of pain sensitivity following the CCI surgery but no differences were observed between these two groups at any time point. Data are shown as mean ± SEM per group per day. # represents a significant difference between the CCI and the OSS+CCI group for the individual time point (^##^*p* < 0.01, ^###^*p* < 0.001). Asterisks indicate a significant difference between the CCI or OSS+CCI group and the OSS as well as the CON group (***p < 0.001).

Four weeks (day 0) before the CCI surgery in the CCI and OSS+CCI animals (performed at day 28), at the beginning of the stress paradigm, we started to assess the cold sensitivity in all groups (Figure 2A). The measurements were carried out once a week (days 0, 7, 14, 21, 27). Paw lifts could be observed in none of the four groups indicating that there was no effect of the social stress itself on thermal pain sensitivity in the OSS and OSS+CCI groups.

The CON and the OSS groups did not display any cold hyperalgesia throughout the observation period. The groups undergoing CCI at day 28 (CCI and OSS+CCI) developed increasing cold sensitivity throughout the next 21 days of observation. However, the extent of paw lifts differed between the two groups at the different time points. Whereas shortly after surgery (day 32) the CCI animals exhibited a statistically significant higher cold sensitivity compared to the OSS+CCI group (5.5 ± 0.6 vs. 3.5 ± 0.6; *p* = 0.0052) they were significantly less sensitive at the end of the experiment at day 49 compared to OSS+CCI (11.2 ± 0.8 vs. 14.3 ± 1.3; *p* < 0.0001). However, as can be seen in Figure 2A, a “switch” in sensitivity seemed to take place 7–14 days after the surgery (day 35: CCI 7.6 ± 0.7, OSS+CCI 6.1 ± 0.7; day 38: CCI 8.4 ± 1.1, OSS+CCI 8.2 ± 0.8; day 42: CCI: 8.9 ± 1.5; OSS+CCI: 9.8 ± 0.9).

Obviously, the cold sensitivity of the CCI and OSS+CCI rats was significantly higher compared to CON and also to OSS animals after the CCI surgery (*p* < 0.0001 for all time points).

### Onset and Maintenance of Mechanical Hypersensitivity Following the CCI Surgery and Stress Protocol

Throughout the observation period and as displayed in Figure 2B, the CON and OSS groups did not display any differences in mechanical sensitivity as compared to basal levels (values are given in Table 3).

**Table 3:** **Von Frey thresholds—data presented as % of baseline for each group**.

Group/Days	0	7	14	21	27	32	35	38	42	49
CON	98.6 ± 1.8	101.7 ± 2.9	95.6 ± 2.5	101.4 ± 2.4	95.8 ± 3.8	99.2 ± 3.7	96.2 ± 3.4	102.8 ± 6.8	100.9 ± 5.0	102.9 ± 5.0
OSS	101.8 ± 2.0	100.6 ± 2.2	103.1 ± 4.9	100.5 ± 2.7	106.1 ± 4.4	100.2 ± 3.1	103.4 ± 3.5	98.3 ± 3.1	97.5 ± 3.3	107.6 ± 4.8
CCI	96.3 ± 2.7	101.3 ± 1.4	98.9 ± 3.0	104.3 ± 2.5	103.3 ± 2.5	19.4 ± 3.0	16.5 ± 1.5	15.4 ± 1.2	21.1 ± 4.1	19.3 ± 2.1
OSS+CCI	100.5 ± 3.0	100.2 ± 5.2	95.4 ± 3.8	99.9 ± 3.4	104.2 ± 3.7	28.8 ± 9.8	17.4 ± 1.6	17.2 ± 2.1	19.3 ± 1.6	17.7 ± 2.1

The CCI and OSS+CCI groups showed a strong increase in pain sensitivity from 4 days post-surgery (day 32) until the end of the experiment on day 49. We did however not find any significant differences between these two groups.

Statistical differences were only observed from day 32 to day 49 (*p* < 0.001) between the CON and OSS groups on one hand compared to the CCI and OSS+CCI rats on the other hand.

### Impact of Ongoing Social Stress and CCI Surgery on Spinal Glial Cell Activation

**Figure 3:**
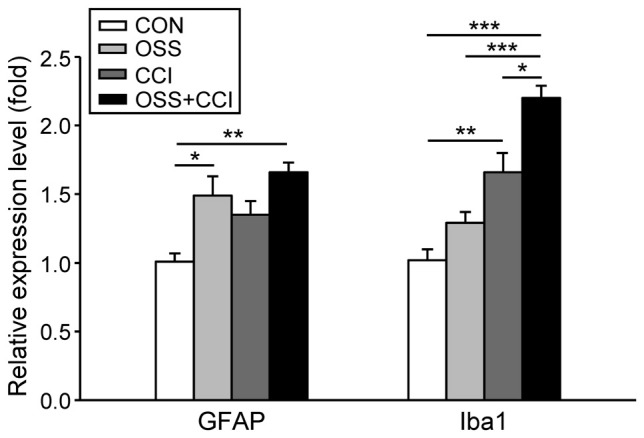
**Influence of social stress and CCI on the mRNA expression of markers for the activation of astrocytes (GFAP) and microglia (Iba1) in the ipsilateral spinal cord levels L4/L5 at day 49**. Data are expressed as relative expression level (fold) of CON (control group = 1, white column) and are shown as mean ± SD (*n* = 6 per group). **Left**: GFAP mRNA expression was increased only in the groups subjected to chronic social stress: OSS (light gray column) and OSS+CCI (constriction injury under social stress conditions, black column), but not in the group that solely underwent CCI (dark gray column). **Right**: Iba1 mRNA expression was increased in the group subjected to constriction injury (CCI) and in the OSS+CCI group where stress enhanced the effect of the constriction injury. No effect was noticeable in the stress group OSS. *p < 0.05, **p < 0.01, ***p < 0.001.

We examined the spinal mRNA expression of two glial cell markers, GFAP for astrocytes and Iba1 for microglia at the end of the experiment at day 49, when the cold sensitivity was highest in CCI and OSS+CCI (Figure 3). We aimed to see if chronic social stress was modifying the neuropathy-mediated activation. In this respect, we focused on changes in the mRNA expression in the right spinal cord levels L4/L5 corresponding to the side of CCI surgery. For all mRNA expression experiments, the ipsilateral spinal cord of the CON group served as control (relative expression level = 1) and the expression levels of the three treatment groups were expressed as fold of CON. For statistical analysis, we compared the expression levels of the experimental groups OSS, CCI and OSS+CCI to CON and the obtained *p*-values are given together with the respective means.

Regarding the activation of astrocytes, there was a significant upregulation in the OSS group (1.49 ± 0.39; *p* = 0.050), a small but not significant increase in the CCI rats (1.35 ± 0.28; *p* = 0.237) and an even more distinct increase in the OSS+CCI animals (1.66 ± 0.12; *p* = 0.006). These data exhibit an amplification of CCI-mediated astrocyte activation under conditions of OSS.

In the case of microglial activation we observed a different pattern. The stress protocol alone did not impact the spinal Iba1 mRNA expression (1.3 ± 0.21; *p* = 0.434) while CCI led to a significant activation of microglia (1.66 ± 0.41; *p* = 0.008). CCI injury under conditions of stress in the OSS+CCI group, however, amplified the mRNA expression of the microglial marker (2.20 ± 0.25; *p* < 0.001). Statistical analysis revealed also significant differences between OSS and OSS+CCI (*p* < 0.001) as well as between CCI and OSS+CCI (*p* = 0.03).

### Spinal mRNA Expression of the Pro-Inflammatory Cytokines IL-1β and IL-6 Following CCI and Chronic Social Stress

**Figure 4:**
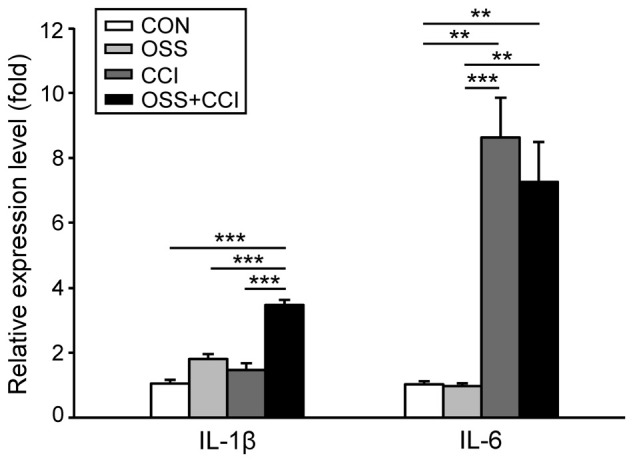
**mRNA expression of the pro-inflammatory cytokines IL-1β and IL-6 in the ipsilateral spinal cord segment L4/L5 following the chronic social stress protocol (OSS, light gray column), CCI (dark gray column) or the combination of both models (OSS+CCI, black column) at day 49**. Data are expressed as relative expression level (fold) of CON (control group = 1, white column) and are shown as mean ± SD (*n* = 6 per group). **Left**: IL-1β mRNA expression was significantly enhanced only in the OSS+CCI animals. **Right**: IL-6 mRNA expression was greatly increased in animals that underwent nerve injury (CCI and OSS+CCI). Social stress alone (OSS) and in combination with CCI (OSS+CCI) had no (additional) effect on the relative expression level of IL-6. **p < 0.01, ***p < 0.001.

In this experiment, we observed a slight, but not significant increase in the spinal mRNA expression of IL-1β in the stress (1.81 ± 0.42; *p* = 0.068) and CCI (1.47 ± 0.58; *p* = 0.503) groups (Figure 4). The combination of CCI and OSS however ended up with a pronounced and highly significant upregulation of this pro-inflammatory cytokine (3.47 ± 0.45; *p* < 0.001). Furthermore there was a statistically significant increase of IL-1β mRNA in the OSS+CCI group as compared to OSS (*p* < 0.001) and CCI (*p* < 0.001).

Regarding IL-6, we did not find any impact of the chronic social stress paradigm on the spinal mRNA expression (0.97 ± 0.25; *p* = 1.00; Figure 4). In contrast, the expression was highly upregulated following CCI surgery (8.63 ± 3.42; *p* < 0.001). Combination of constriction injury with social stress did not further enhance the expression level (7.26 ± 3.45; *p* = 0.003). Additionally, we found statistically significant differences between OSS and CCI (*p* < 0.001) as well as between OSS and OSS+CCI (*p* = 0.002) but not for CCI vs. OSS+CCI (*p* = 0.82).

### Effect of CCI and Chronic Social Stress on the mRNA Expression of the Neurotrophic Factors GDNF and NGF

**Figure 5:**
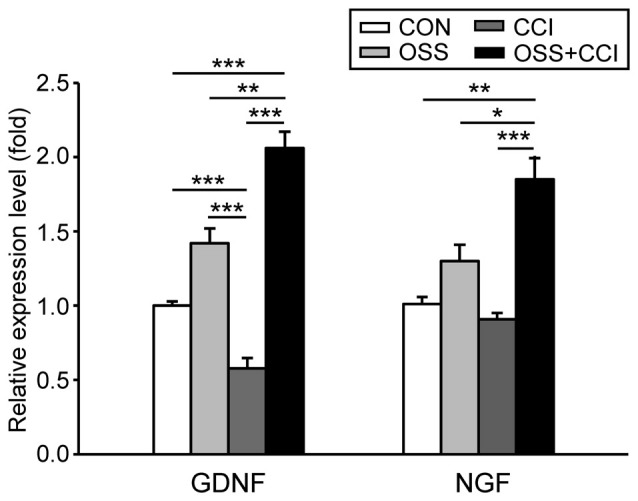
**Effect of chronic social stress and nerve injury on the mRNA expression of the neurotrophic factors glial cell line-derived neurotrophic factor (GDNF) and nerve growth factor (NGF) in the ipsilateral spinal cord level L4/L5 at day 49**. Data are expressed as relative expression level (fold) of CON (control group = 1, white column) and are shown as mean ± SD (*n* = 6 per group). **Left**: GDNF mRNA expression was slightly but not significantly increased in the group subjected to OSS (light gray column) and significantly downregulated after nerve injury (CCI, dark gray column). However, after CCI under conditions of social stress (OSS+CCI, black column) the relative expression levels of GDNF mRNA were significantly upregulated. **Right**: NGF mRNA expression did neither change after exposure to chronic social stress (OSS) nor following CCI surgery (CCI). Nerve injury under chronic social stress (OSS+CCI) did however lead to a significant upregulation of NGF mRNA expression. *p < 0.05, **p < 0.01, ***p < 0.001.

Chronic social stress during adolescence/early adulthood slightly but not significantly increased the spinal expression level of GDNF mRNA (1.42 ± 0.29; *p* = 0.139; Figure 5), whereas CCI surgery significantly decreased the mRNA level (0.58 ± 0.21; *p* < 0.001). The stress component in the OSS+CCI group greatly outweighed the decreasing effect of the constriction injury and finally led to a two-fold increase (2.06 ± 0.32; *p* < 0.001) in the mRNA expression level of GDNF. Statistical analysis also unravelled significant differences between OSS and CCI (*p* < 0.001), OSS and OSS+CCI (*p* = 0.004), as well as between CCI and OSS+CCI (*p* < 0.001).

The expression of spinal NGF mRNA was unaffected by the chronic social stress procedure (1.30 ± 0.32; *p* = 0.515) and by the CCI surgery (0.91 ± 0.11; *p* = 0.967; Figure 5). However, the combination of both manipulations resulted in a significant upregulation of the NGF mRNA (1.85 ± 0.45; *p* = 0.004). The expression level was also significantly different from OSS (*p* = 0.044) and CCI (*p* < 0.001).

### Implication of the Glutamatergic System in CCI and Chronic Social Stress Mechanisms

To investigate the involvement of the glutamatergic system in the development and maintenance of neuropathic pain and in the processing of chronic social stress, we measured the mRNA expression of NR1 and NR2a, two common subunits of the NMDA receptor and of EAAT3, a glial transporter of glutamate.

**Figure 6:**
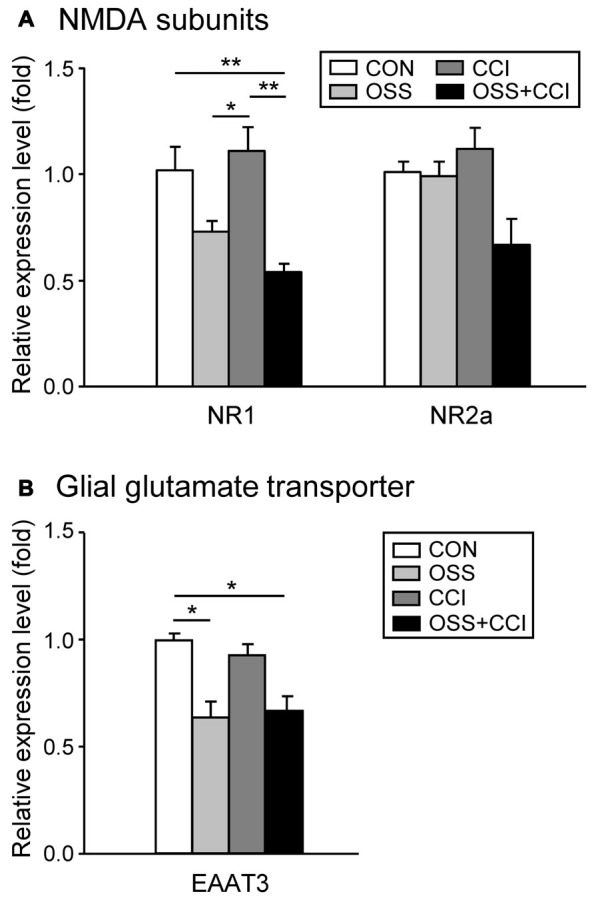
**Modifications of the glutamatergic system following a chronic social stress protocol and/or CCI surgery**. The mRNA expression of the two NMDA receptor subunits NR1 and NR2a and the glial glutamate transporter EAAT3 were measured in the ipsilateral spinal cord segment L4/L5 following either chronic social stress (OSS, light gray column), or CCI (dark gray column), or the combination of both conditions (OSS+CCI, black column), at day 49. Data are expressed as relative expression level (fold) of CON (control group = 1, white column) and are shown as mean ± SD (*n* = 6 per group). **(A)** The NR1 mRNA expression was slightly decreased by social stress (OSS), not affected by the CCI surgery (CCI), but significantly downregulated after nerve injury under stress conditions (OSS+CCI). The relative expression level of NR2a did not significantly change in the three experimental groups, although a small decrease could be observed for the OSS+CCI group. **(B)** The EAAT3 mRNA expression was significantly downregulated in the two groups subjected to chronic social stress: OSS and OSS+CCI. CCI alone had no effect on the relative expression level of the glial glutamate transporter. *p < 0.05, **p < 0.01.

The spinal NR1 mRNA expression was slightly, but not significantly decreased in the OSS group (0.73 ± 0.14; *p* = 0.243; Figure 6A). The CCI surgery alone had no effect on NR1 expression levels (1.11 ± 0.31; *p* = 0.908) but induction of neuropathy under stress conditions caused a significant downregulation of NR1 mRNA transcription (0.54 ± 0.10; *p* = 0.020) exceeding the decrease related to stress alone. This reduction in the OSS+CCI group turned out to be also statistically significant compared to CCI (*p* = 0.002). Although the expression level in the OSS group was not significantly different from the CON, it differed from the CCI group (*p* = 0.039).

Regarding the NR2a subunit of the NMDA receptor, we did not find any significant alteration in the mRNA expression, although a small decrease was apparent for the OSS+CCI group.

OSS significantly downregulated the spinal mRNA expression of EAAT3 (0.64 ± 0.21; *p* = 0.046; Figure 6B). On the other hand, neuropathy had no impact on the regulation of this transporter (0.93 ± 0.13; *p* = 0.954). In the OSS+CCI group the impact of the social stress predominated and led likewise to a significant downregulation of EAAT3 mRNA in the ipsilateral spinal cord level L4/L5 (0.62 ± 0.21; *p* = 0.033). Glutamate might hence have accumulated in the synaptic cleft, reinforcing synaptic transmission.

## Discussion

In the present study the major finding resulting from the combination of preclinical models of neuropathic pain and psychosocial stress was a switch in cold pain sensitivity from initially dampened to exacerbated allodynia. For mechanical sensitivity no differences were observed between stressed and non-stressed neuropathic rats. Biochemical results revealed an activation of astrocytes in a stress condition but also of microglia under conditions of combined stress and neuropathic pain, a differential regulation of pro-inflammatory cytokine mRNA, IL-1β being more prone to the combination of stress and neuropathy while the expression of IL-6 was mainly enhanced in neuropathic conditions, a synergistic effect of stress and neuropathy regarding the expression of neurotrophic factor mRNA, and finally a decrease of the transporter EAAT3 and NMDA receptor subunit NR1 in the stress group and in the group exposed to both stress and chronic constriction injury.

### Selection of the Chronic Social Stress Model

As mentioned in the introduction, our goal was to focus on the impact of psychosocial stress on neuropathic pain sensitivity. While some studies using psychological stressors observed an enhancement of pain sensitivity (Rivat et al., 2007; Burke et al., 2013), others failed to identify similar effects (Bravo et al., 2012, 2013). These divergent findings may partly be related to methodological differences with respect to the approaches chosen for stress induction.

In the present study, we did therefore aim at using a rodent model that would as closely as possible match ongoing psychosocial stress situations that humans are commonly exposed to. The model we chose was introduced in mice by Schmidt et al. (2007) and derived from a model initially developed in rats by Mormède et al. (1990). Briefly, the group composition of male rat cage mates was changed in regular intervals starting at the age of 4 weeks until the end of the experimental protocol at 11 weeks. The stress hence started during the adolescence period, meaning in puberty, a period of life that is highly adaptive and during which substantial remodeling occurs in areas involved in emotional and learning processes (Tsoory et al., 2007). Furthermore, this period is also of much importance regarding social interaction and acquisition of social skills that will induce stability in adulthood (Sachser et al., 1998). Hence stressing the animals in this precise period when the behavior and neuroendocrine system are fine-tuned can have important repercussions in adulthood. Finally, contrary to many stress models used in the literature, this ongoing social stress is continuous (most of the stressors are only applied during some time of the day), inescapable for the animal, applicable to a large number of animals in order to have an adequate rotation schedule and less offensive than most other models such as social defeat or chronic unpredictable stress.

In order to have objective markers of the impact of stress we measured plasmatic corticosterone levels once a week during all the experiment. However we were not able to demonstrate any statistical differences between the groups (data not shown). This is in accordance with several studies (Norman et al., 2010b; Shi et al., 2010) and can be explained by an adaptation of the HPA axis over time. Regarding the body weight of the animals also, it is known that chronic stress can induce a reduction (Shi et al., 2010; Bravo et al., 2012). We did however not observe any differences between the four groups. In this respect Schmidt et al. (2007) have claimed that the animals have a different weight regulation during the adolescent period where the stress paradigm has been started and that during this period the regulation of growth is less influenced by environmental factors. In addition adolescent animals might compensate more easily for weight loss than adult animals.

Finally, we only used male rats. It is however established that sex can have an influence on pain sensitivity (for review, see Mogil, 2012) but also on stress impact (Burke et al., 2013). We did not observe any stress model-related differences in mechanical sensitivity for the male rats we used, in accordance with Burke et al. (2013) who did however observe a reduction in mechanical pain threshold and a sensitization of the contralateral paw in female Wistar rats. These findings point to the importance of considering sex-related effects.

### Development and Maintenance of Cold and Mechanical Hypersensitivity

Regarding the assessment of pain sensitivity, the cold plate test has been shown to reliably reveal cold hypersensitivity, a characteristic of CCI-induced neuropathic pain (Berrocoso et al., 2011). We chose this paradigm because exacerbated cold sensitivity is more often observed than heat hypersensitivity in clinical neuropathic conditions (Attal et al., 2008). The test was performed regularly from the beginning of the stress protocol (day 0) to 21 days after the eventual CCI surgery (day 49). Before CCI, no paw lifting was observed for any group, confirming that we observed neuropathy-related allodynia. CCI and OSS+CCI rats started to display cold plate related pain behavior a few days post-surgery, with the stress seeming to have a protective effect in the OSS+CCI group as compared to the CCI group.

Acute and chronic stressors may differentially affect pain. Under acute conditions, “stress-induced analgesia” (SIA) is commonly observed (for review, see Amit and Galina, 1998) while chronic stress may more regularly be accompanied by hyperalgesic states (Suarez-Roca et al., 2006; Jennings et al., 2014). Our results from days 32 and 35 could reflect SIA. At day 38, the two groups had equivalent sensitivities when finally, at day 49, we observed a switch, the OSS+CCI rats displaying a hypersensitivity to cold as compared to the CCI group. This phase could correspond to the SIH phenomenon that to our knowledge has not yet been described for neuropathic pain conditions. While these results may seem contradictory to observations by Aghajani et al. (2012) who did not detect any impact of a social instability model on chronic pain in mice, other studies using cold stress (Imbe et al., 2006) or social defeat (Marcinkiewcz et al., 2009) demonstrated increased nociceptive responses. Other stress paradigms such as repeated swim stress (Suarez-Roca et al., 2006; Quintero et al., 2011) or chronic restraint stress (da Silva Torres et al., 2003; Bardin et al., 2009) also ended up with a long lasting hyperalgesia. It should however be kept in mind that these physical stressors may not match our conditions of ongoing social stress. Ultimately, the described reversion in behavioral sensitivity came quite as a surprise to us at this time point and experiments using a longer observation period than the initially scheduled 49 days are required to confirm the significance of this effect.

Concerning our failure to identify differences in mechanical sensitivity between CCI and OSS+CCI groups, it is interesting to note that Bardin et al. (2009) as well as Bravo et al. (2012, 2013) reported similar findings in a model of chronic mild stress and in a model of social isolation. Whereas the temperature of the cold plate test (5°C) used in the present study quite selectively stimulates cold nociceptors of the C-fiber type (Simone and Kajander, 1996), the von Frey filaments may activate a larger range of nociceptors belonging both to the C- and A-delta fiber categories (Martin et al., 1997). In the framework of the present study, it could hence be speculated that the ongoing psychosocial stress primarily affected the processing of C-fiber rather than A-delta fiber mediated nociceptive processing. In line with this reasoning, there is evidence for an interaction of stress hormones with nociceptive C fibers (Chen and Levine, 2005). Selective pharmacological inhibition of C- but not A-delta fiber-mediated nociception has recently been described by You et al. (2009). Alternatively, stress-mediated effects may have depended on factors like stimulus modality, sensory transduction, stimulation surface and duration. It should also be noted that for both stimulation modalities we did not observe any neuropathic pain-related effects at the contralateral paw, findings that are congruent with earlier studies (Pitcher and Henry, 2004; Benbouzid et al., 2008; Zeng et al., 2008).

### Glial Cell Activation and Pro-Inflammatory Cytokine Expression

Regarding astrocytes and microglia, we observed different patterns of activation pointing to distinct roles in the mediation of pain and stress signals. In agreement with previous investigators (Lambert et al., 2000), we noticed a stress-related enhancement of GFAP mRNA expression. This expression was similar in the OSS+CCI group, indicating that astrocyte activation might primarily have depended on stress- rather than on neuropathy-related mechanisms. While the CCI group displayed an activation of microglia, the greatest increase in mRNA expression was observed for OSS+CCI. The finding that microglial activation is usually accompanied by a behavioral hypersensitivity (Watkins et al., 2001; Zhuo et al., 2011) is in accordance with the OSS+CCI group expressing more pronounced cold hyperalgesia at day 49.

Microglial and astrocytic activation induces the release of pro-inflammatory cytokines (for review, see Watkins et al., 2001). This is in agreement with the enhanced expression of IL-1β mRNA noticed in the OSS+CCI group. In addition, psychosocial stress-related microglia activation has previously been observed in different brain areas such as the hippocampus, amygdala and prefrontal cortex, leading to the production of IL-1β (Wohleb et al., 2011; Hinwood et al., 2012). Also, in a model of isolation stress combined with a model of cardiac arrest surgery (Norman et al., 2010b), and under conditions of maternal deprivation combined with spinal nerve ligation (SNL) neuropathy model (Burke et al., 2013), mRNA for Il-1β was shown to be upregulated in the hippocampus. The expression of IL-6 mRNA was significantly increased to a similar extent in the CCI and OSS+CCI groups and thus seemed to be selectively implicated in inflammatory processes inherent to CCI-related neuropathic pain. Our finding is in accordance with hippocampus-related data from Burke et al. (2013) but contradictory to previous results describing a stress-mediated release of this cytokine in mice (Norman et al., 2010a; Aghajani et al., 2012). This could be due to the use of different stress paradigms or to species differences. The fact that in our hands IL-1β mRNA expression was most pronounced in the OSS+CCI rats may point to a combined induction by stress- and pain-related mechanisms.

### Implication of Neurotrophic Factors

Our major observation concerning the spinal mRNA expression pattern of NGF and GDNF was that stress led to enhanced levels, while a diminished expression was exhibited by the CCI group. In the OSS+CCI rats, the respective expression changed into the same direction as for the stress group, albeit to a more pronounced extent.

GDNF is involved in cell survival, differentiation and migration. Its serum level is reduced in patients with major depression (Miller, 2011) and decreased in the dorsal root ganglia (DRG) after nerve injury (Nagano et al., 2003). It has also been demonstrated that GDNF has a beneficial effect on neuropathic pain. A continuous administration ameliorated pain responses and suppressed pain behavior following SNL or CCI (Boucher et al., 2000; Nagano et al., 2003; Chou et al., 2014). These data are in accordance with our observations in the CCI group. The most important increase noticed in the OSS+CCI group at day 49, when the respective animals were hypersensitive to the cold plate, seems to indicate that GDNF could have an additional stress-related function that could be more pronounced than the neuropathy-related effects *per se*. In addition, an increase in GDNF could result from the activation of astrocytes (Bian et al., 2012). This observation could corroborate our data, the GFAP marker mRNA displaying the same pattern of expression as GDNF.

Regarding NGF, controversial findings have been described. While some studies demonstrated a stress-related decrease of NGF mRNA expression in DRG or hippocampus (Nagano et al., 2003; Alfonso et al., 2004), others revealed an increase under such conditions (for review, see Miller, 2011). On the other hand, NGF has been shown to be a major contributor to the production of inflammatory hyperalgesia and maintenance of neuropathic pain (Matsuura et al., 2013). An increased expression has been observed in a CCI model of neuropathic pain (Nagano et al., 2003; Santos et al., 2012) and a single injection of NGF has been shown to induce hyperalgesia (Lewin et al., 1993). In the present study we observed almost no change in the mRNA expression of NGF in the CCI group. We did however find an increase in the OSS group that was even more pronounced in the OSS+CCI group. In our setting, NGF could hence have been more implicated in stress mechanisms than in pain processing itself. The fact that we observed comparable bilateral patterns of mRNA expression for GDNF and NGF (data not shown) seems to support this hypothesis.

Since it has also been shown that the pro-nociceptive effect of NGF is blocked by MK-801, an NMDA receptor antagonist (Herzberg et al., 1997), we investigated the implication of glutamatergic transmission via the mRNA expression of a glutamate transporter and two subunits of the NMDA receptor.

### Involvement of the Glutamatergic System

Regarding the glutamate transporter EAAT3 and the NR1 subunit of the NMDA receptor, comparable patterns of mRNA expression, namely a downregulation, were noticed in the OSS and OSS+CCI groups.

It has been shown that the glutamatergic system has a predominant implication in the relationship between the HPA axis, activated by stress mechanisms, and neuropathic pain (Le Coz et al., 2014a). The NMDA receptor may be implicated both in neuropathic pain and in stress mechanisms (Gould and Tanapat, 1999). Repeated cold stress leads to an enhanced sensitivity of the NMDA receptor and to a facilitation of glutamate release in the spinal cord and hippocampus (Imbe et al., 2006; Quintero et al., 2011). This is also true in conditions of neuropathic pain (Wang et al., 2005). We did however observe the opposite effect for OSS and no change for CCI. Since the mRNA expression of NR1 was even more decreased in the OSS+CCI rats, the stress-related mechanisms might have overruled the nociception-related ones.

To our knowledge, there are no published data on transporter expression in a context of psychosocial chronic stress. Our observations seem to indicate a more important impact of the chronic social stress than of the CCI procedure on the spinal expression of EAAT3 mRNA. One study showed that a repeated restraint stress enhanced glutamate release and uptake (Imbe et al., 2006). This increase can be interpreted as an augmentation in the expression of transporters, but in the present study we observed the opposite effect regarding EAAT3. Gosselin et al. (2010) also observed a decrease in glutamate transporter (EAAT1) in the spinal astrocytes of rats undergoing maternal separation. These discrepancies may be related to the use of different stress paradigms.

As previously described, a link is possible between the neurotrophic factor NGF and the NMDA receptor. An activation of NMDA receptors will induce an increase of NGF expression (Herzberg et al., 1997). In the OSS+CCI group we did however observe an increase of the NGF mRNA expression and a decrease of the NR1 subunit of the NMDA receptor. Also, we did not detect any differences regarding the mRNA expression of the NR2a subunit of the NMDA receptor. These discrepancies may be due to the stress and pain models used in the present study. Other subunits that could be involved should be considered in further investigations.

### Concluding Remarks

Our exploratory study confirms that exposure to long lasting social stress starting during adolescence may enhance neuropathic pain-related cold sensitivity in adulthood. At the level of the spinal cord, the combination of psychosocial stress and neuropathic pain may have an important incidence on glial cell activation, on the release of pro-inflammatory cytokines and of neurotrophic factors and to a lesser extent on glutamatergic transmission. Further studies will have to include protein level measurements. For correlative purposes, the observation period should be extended and the measurements of biochemical markers should include earlier time points preceding and including the observed switch of pain sensitivity. The potential role of ongoing stress on descending pain control pathways will also have to be considered.

## Author Contributions

G-MLC, FA and UH derived the original design of the study; G-MLC, UH and JG acquired, analyzed and interpreted the data, G-MLC drafted the original manuscript; UH, FA and JG revised the manuscript. All authors read and approved the final manuscript.

## Funding

This work was supported by an Internal Project Grant of the University of Luxembourg (F3R-INS-PUL-11STPA). The funder had no role in study design, data collection and analysis, decision to publish, or preparation of the manuscript.

## Conflict of Interest Statement

The authors declare that the research was conducted in the absence of any commercial or financial relationships that could be construed as a potential conflict of interest.
